# Evaluation of the Prevalence, Regional Phenotypic Variation, Comorbidities, Risk Factors, and Variations in Response to Different Therapeutic Modalities Among Indian Women: Proposal for the Indian Council of Medical Research–Polycystic Ovary Syndrome (ICMR–PCOS) Study

**DOI:** 10.2196/23437

**Published:** 2021-08-27

**Authors:** Mohd Ashraf Ganie, Subhankar Chowdhury, Vanita Suri, Beena Joshi, Prasanta Kumar Bhattacharya, Sarita Agrawal, Neena Malhotra, Rakesh Sahay, Roya Rozati, Puthiyaveettil Khadar Jabbar, Vishnubhatla Sreenivas, Mukesh Sriwastva, Imtiyaz Ahmad Wani, Shalini Singh, Radhey Shyam Sharma

**Affiliations:** 1 Department of Endocrinology & Clinical Research Sher-i-Kashmir Institute of Medical Sciences Srinagar India; 2 Department of Endocrinology & Metabolism Institute of Postgraduate Medical Education & Research Kolkata India; 3 Department of Obstetrics & Gynaecology Postgraduate Institute of Medical Education and Research Chandigarh India; 4 Department of Operational Research National Institute for Research in Reproductive Health Indian Council of Medical Research Mumbai India; 5 Department of Community Medicine North Eastern Indira Gandhi Regional Institute of Health and Medical Sciences Shillong India; 6 Department of Obstetrics & Gynaecology All India Institute of Medical Sciences Raipur India; 7 Department of Obstetrics & Gynaecology All India Institute of Medical Sciences New Delhi India; 8 Department of Endocrinology Osmania Medical College Hyderabad India; 9 Department of Obstetrics & Gynaecology Maternal Health & Research Trust Hyderabad India; 10 Department of Endocrinology Government Medical College Thiruvananthapuram India; 11 Department of Biostatistics All India Institute of Medical Sciences New Delhi India; 12 Department of Endocrinology and Metabolism All India Institute of Medical Sciences New Delhi India; 13 Reproductive Biology and Maternal Health, Child Health Indian Council of Medical Research New Delhi India

**Keywords:** polycystic ovary syndrome, prevalence, metabolic aberrations, community pool, therapeutic modalities, India, metabolic dysfunction, phenotypic variations, ovarian morphology, PCOS epidemiology

## Abstract

**Background:**

There is scanty data in India on polycystic ovary syndrome (PCOS) from several small, undersized, convenience-based studies employing differing diagnostic criteria and reporting varied regional prevalence. It is difficult to draw clear-cut conclusions from these studies; therefore, the present multicentric, well-designed, large-scale representative countrywide epidemiological study on PCOS across India was conceived with the aim to generate the actual prevalence rates of PCOS in India with a total sample size of approximately 9000 individuals.

**Objective:**

The primary objectives of the study are to estimate the national prevalence of PCOS in India and the burden of comorbidities and to compare the variation in efficacy of standard therapeutic modalities for metabolic dysfunction in women with PCOS.

**Methods:**

This multicentric umbrella study consists of three different substudies. Substudy 1 will involve recruitment of women aged 18-40 years using a multistage sampling technique from randomly selected polling booths across urban and rural areas to estimate national prevalence, phenotypic variation, and risk factors among regions. Substudy 2 involves recruitment of subjects from the community pool of substudy 1 and the institutional pool for quantitation of comorbidities among women with PCOS. Substudy 3, an interventional part of the study, aims for comparison of variation in efficacies of common treatment modalities and will be conducted only at 2 centers. The eligible consenting women will be randomized in a 1:1 ratio into 2 arms through a blinding procedure. All these women will undergo clinical, biochemical, and hormonal assessment at baseline and at 3 and 6 months. The data generated will be analyzed using the reliable statistical software SPSS (version 26).

**Results:**

The study is ongoing and is likely to be completed by April 2022. The data will be compiled and analyzed, and the results of the study will be disseminated through publications.

**Conclusions:**

The Indian Council of Medical Research–PCOS study is the first of its kind attempting to provide accurate and comprehensive data on prevalence of PCOS in India.

**Trial Registration:**

Clinical Trials Registry–India CTRI/2018/11/016252; ctri.nic.in/Clinicaltrials/pmaindet2.php?trialid=26366

**International Registered Report Identifier (IRRID):**

DERR1-10.2196/23437

## Plain English Summary

Polycystic ovary syndrome (PCOS) is a multifactorial disorder with unknown etiology and numerous clinical manifestations in reproductive-aged women around the world. The metabolic abnormalities linked to this syndrome include type 2 diabetes, insulin resistance (IR) obesity, abnormal glucose tolerance (AGT), and cardiovascular diseases (CVD). The sketchy data and escalating prevalence of PCOS and associated comorbidities among Indian women led to organization of a multidisciplinary brainstorming meeting held September 11, 2014, at Indian Council of Medical Research (ICMR) headquarters, and the ensuing recommendations suggested formulation of the ICMR–PCOS Task Force to conduct a multicentric, multiphase study among Indian women with PCOS.

This is a multicentric, multispecialty study with a common comprehensive predesigned protocol involving 10 centers located in the 6 zones of the country. The women from the community (rural and urban) will be screened using a validated screening questionnaire, after obtaining a written informed consent from the subjects. The proforma will capture all details about menstrual cyclicity, acne, hirsutism, socioeconomic status, family history of PCOS, etc. The national prevalence will be calculated from the community pool for substudy 1. All biochemical and hormonal analysis will be done by standard assays using similar kits, reagents, and equipment. Ultrasonography (US) including magnetic resonance imaging (MRI) of the abdomen will be performed using a common approved protocol. The various comorbidities such as AGT, nonalcoholic steatohepatitis, and sleep apnea will be evaluated from both the pools (ie, hospital cohort and community cohort in substudy 2). The interventional randomized double-blind trial (ie, oral contraceptive pills [OCPs] vs metformin) will be conducted at only 2 centers for substudy 3. Apart from this, a knowledge, attitude, and practices survey of service providers in the public health system will be conducted to understand the training needs and gaps in the health system addressing PCOS management.

## Introduction

### Overview

Polycystic ovary syndrome, affecting approximately 116 million women worldwide, is now considered the commonest endocrinopathy of reproductive-aged women [1,2]. Stein and Leventhal initially described it as the association of bilateral ovaries with amenorrhea in 1935 [3,4]. Now, its association with a constellation of metabolic aberrations including obesity, AGT, IR, non-alcoholic fatty liver disease (NAFLD), metabolic syndrome, sleep apnea, stroke, CVD, neuropsychiatric comorbidities, microalbuminuria, mitogenesis, etc, in addition to cosmetic and reproductive dysfunction, is increasingly being recognized [5-9].

Global prevalence of PCOS among geographical zones is reported differently as 5%-10% in developed countries, 5%-30% in the United States, and 3.7%-22.5% across India [10-13]. Since there is no specific established treatment protocol available, early diagnosis and management is likely to reduce the risk of long-term complications of PCOS. The etiology of the disorder is being actively investigated; various reports speculate on the environmental and genetic factor interactions responsible [14-16]. Various small, regional, poorly designed studies using varying diagnostic criteria suggest a high prevalence of PCOS in India, seemingly running parallel to the epidemic of noncommunicable diseases, especially type 2 diabetes. There are no systematic representative data on the prevalence of PCOS or burden of its comorbidities among Indian women to date [17-31]. The existing data hint at the potential for large-scale epidemiological studies on PCOS across India to generate the actual prevalence and picture of the disease in the community. Therefore, there is an immense need to generate nationwide data on the prevalence of PCOS, including its comorbidities, besides any variations in phenotypes or treatment responses among Indian women.

To address these issues concerning PCOS among Indian women, ICMR, New Delhi, has taken the initiative to conduct a nationwide multicentric study titled “Evaluation of prevalence, regional phenotypic variation, co-morbidities, risk factors and the variations in response to different therapeutic modalities among Indian women with polycystic ovary syndrome (PCOS): A Multicenter study across India” to quantitate the burden of PCOS and its comorbidities in its phase 1.

### Objectives

The objectives of the proposed study are as follows:

#### Primary Objectives

The primary objectives are to (1) estimate national prevalence, phenotypic variations, and risk factors of PCOS in the community; (2) estimate the burden of comorbidities such as metabolic syndrome, dyslipidemia, cardiovascular complications, NAFLD, psychiatric abnormalities, sleep apnea, AGT including type 2 diabetes, and impairment of quality of life (QOL) among known cases of PCOS; and (3) compare the variation in efficacy of standard therapeutic modalities for metabolic dysfunction in women with PCOS (2 sites, All India Institute of Medical Sciences [AIIMS] and Sher-i-Kashmir Institute of Medical Sciences [SKIMS]).

#### Secondary Objectives

The secondary objective is to assess knowledge and management practices among service providers in the public health sector on PCOS to develop a standard screening and training modules to facilitate management of PCOS at different levels of the public health care system.

### Prerequisites for Initiation of the Study

To begin with, the study required establishment of a PCOS network comprising members from the specialties and departments of endocrinology, dermatology, community medicine, biochemistry, laboratory medicine, pediatrics, internal medicine, obstetrics and gynecology, and other relevant departments based on their expertise and interest in the area of PCOS. In addition, there will be a team of national reviewers and advisors (members of the Task Force Committee) who will guide in formulating the protocol, advising on methods of assessment, and analyzing the data on relevant aspects. In view of the relevance to the project, a cardiologist (for cardiovascular risk assessment), a gastroenterologist (for NAFLD assessment), a radiologist (for devising methodology for ovarian morphology and implications of data interpretation), a psychiatrist (for assessment of psychiatric comorbidity and QOL), a pediatrician (for assessment of prepubertal risk factors and design of interventions), a dermatologist (for hyperandrogenism assessment strategies), a molecular biologist (for genetic and epidermal differentiation complex work-up), and a biostatistician (for sampling designs and final analysis) have been incorporated in the team and committee.

**Figure 1:**
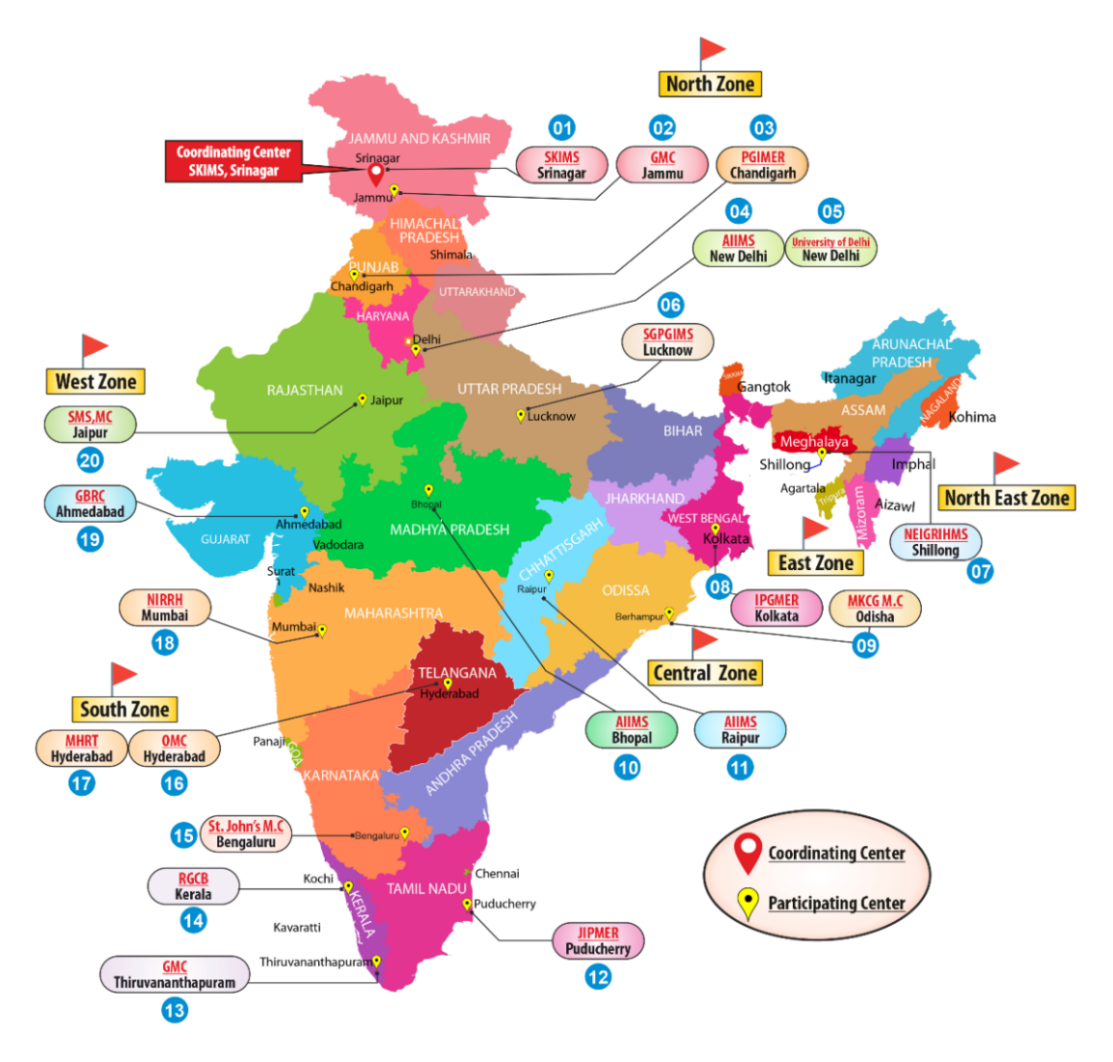
Layout of participating centers. AIIMS: All India Institute of Medical Sciences; GMC: Government Medical College; IPGMER: Institute of Post Graduate Medical Education & Research; MHRT: Maternal Health & Research Trust; NEIGRIHMS: North Eastern Indira Gandhi Regional Institute of Health and Medical Sciences; NIRRH: National Institute for Research in Reproductive Health; OMC: Osmania Medical College; PGIMER: Postgraduate Institute of Medical Education and Research; SKIMS: Sher-i-Kashmir Institute of Medical Sciences.

The following participating centers are involved in the study (Figure 1): (1) Sher-i-Kashmir Institute of Medical Sciences, Srinagar (North); (2) Postgraduate Institute of Medical Education and Research (PGIMER), Chandigarh (North); (3) All India Institute of Medical Sciences, New Delhi (North); (4) ICMR National Institute for Research in Reproductive Health (NIRRH), Seth G S Medical College and King Edward Memorial Hospital, Grant Medical College and JJ Hospital, Mumbai (West); (5) Institute of Post Graduate Medical Education & Research (IPGMER), Kolkata (East); (6) North Eastern Indira Gandhi Regional Institute of Health and Medical Sciences (NEIGRIHMS), Shillong (North East); (7) All India Institute of Medical Sciences, Raipur (Central); (8) Osmania Medical College, Hyderabad (South); (9) Maternal Health & Research Trust, Hyderabad (South); and (10) Government Medical College, Thiruvananthapuram (South).

## Methodological Steps and Study Design

### Overview

The milestone steps to proceed with estimation of national prevalence, phenotypic variation, and risk factors involve a community-based, cross-sectional survey to recruit women belonging to the age group of 18-40 years from both rural (n=500) and urban areas (n=500) across 6 zones (ie, North, South, East, West, North East, Central India). ICMR has always been a leader in developing ethical standards for any human research considering the moral, ethical, and social values and ethos of diverse populations in the country. The proposed study will be conducted according to the Helsinki Declaration of 1975 and stands approved by the Institutional Ethics Committees at all 10 study sites. A written informed consent will be obtained from the participants assuring their understanding of the purpose of the study and obligations and consequences of their participation.

### Detailed Methodology: Substudies 1 and 2

#### Participant Selection

Women aged 18-40 years who are not pregnant or lactating and have been residing in the area for 1 year or more will be included in the study. Women who are known to have PCOS will be recorded for the purposes of PCOS prevalence and will only be evaluated in detail for substudy 2 if they are drug-naïve. Women taking oral contraceptives, steroids, antiepileptics, or other drugs known to interfere in glucose or lipid metabolism will not be evaluated in detail and will be excluded from the study.

#### Substudy 1: Community Pool

The study design is a two-stage, cross-sectional, community-based study.

#### Study Population

Women aged 18-40 years will be recruited using a multistage sampling technique involving urban and rural areas. The polling booths will be selected randomly at the coordinating center by a random selection process. This study will be conducted in 6 zones of the country involving 10 centers that are likely to represent a majority of ethnic groups.

#### Inclusion Criteria

The following are the criteria for inclusion: women aged 18-40 years who are permanent residents of the area (>1 year), willing to participate in the study and sign informed consent, known to have PCOS, and not on treatment.

#### Exclusion Criteria

Pregnant or lactating women and those with cognitive limitations, physical limitations, or both that prevented them from answering the questionnaire will be excluded from the study.

Women with a history of drug intake such as steroids, androgens, oral contraceptives, antiepileptics, or drugs known to interfere in glucose or lipid metabolism will also be excluded.

#### Sampling Design

A multistage cluster sampling technique is being used in this study. The first step in the sampling procedure will be to have representative data from the 6 zones (N, S, E, W, NE, and Central) of the country (Figure 1). The participating institutions from 6 zones were selected in view of location, logistics, feasibility, and the investigator profile. One urban and one rural Vidhan Sabha constituency near each institution will be selected. The choice of selection of districts and the constituencies will be given to the principal investigators at each site in order to make the study execution simpler. A rural district would mean 70% of subjects pursuing agriculture activities, while urban would mean that less than 30% have agriculture as the source of income.

**Figure 2:**
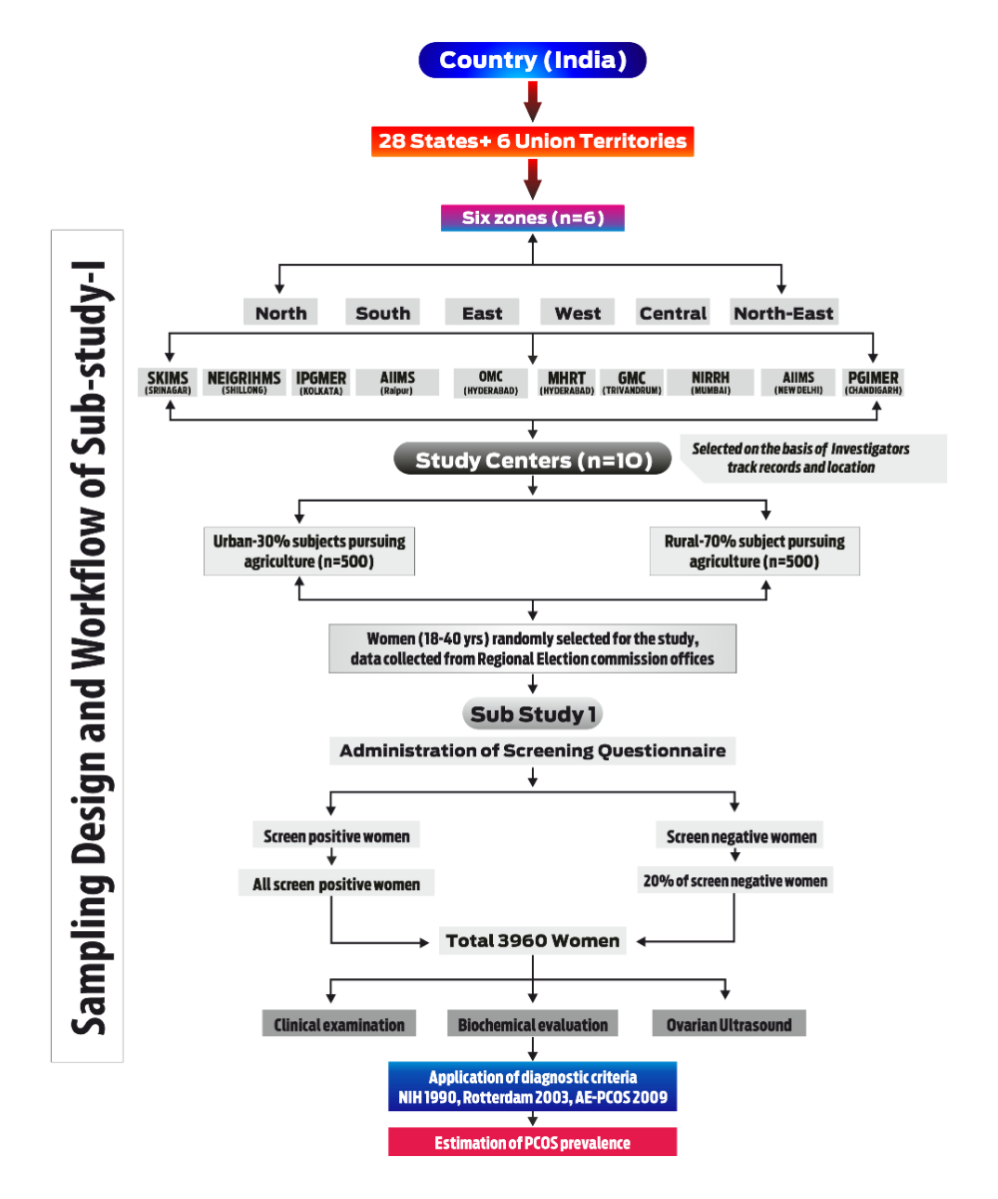
Flowchart for sampling design and workflow of the study. AE-PCOS: Androgen Excess and Polycystic Ovary Syndrome; AIIMS: All India Institute of Medical Sciences; GMC: Government Medical College; IPGMER: Institute of Post Graduate Medical Education & Research; MHRT: Maternal Health & Research Trust; NEIGRIHMS: North Eastern Indira Gandhi Regional Institute of Health and Medical Sciences; NIH: National Institutes of Health; NIRRH: National Institute for Research in Reproductive Health; OMC: Osmania Medical College; PCOS: polycystic ovary syndrome; PGIMER: Postgraduate Institute of Medical Education and Research; SKIMS: Sher-i-Kashmir Institute of Medical Sciences.

After the constituencies are selected, the entire list of polling booths will be obtained from the Regional Election Commission offices. Polling booths from rural or urban constituencies will be selected to fulfill the required sample size using a random number table so that each polling booth has an equal chance to get selected. This procedure will be carried out at the coordinating center (SKIMS, Srinagar) by a third party not directly involved in the study. Next, the sites will procure the electoral roll, and voter IDs will be searched for women (aged 18-40 years) to be the potentially eligible subjects. The resulting list of women will be screened using a checklist for eligibility. If a woman is not eligible or refuses to participate in the study, the offer will go to the next woman in the list. If there is more than one eligible woman in a family, the sampling frame will also shift to the next in the voter ID list. The procedure will continue until a total of 1000 women (500 rural and 500 urban) are screened per center (Figure 2).

#### Estimation of Sample Size for Substudy 1

Sample size was calculated, primarily to estimate the prevalence of PCOS in the country. Previously reported prevalence rates of PCOS have varied from 3.7% to 22.3% [32,33]. For the purpose of calculation of sample size, we presume the prevalence of PCOS in India to be 10%. With an assumed prevalence of 10% and an absolute error margin of 1% in a 95% 2-sided confidence interval and making allowance for about 20% refusals or nonresponse, we need to target a total of 4500 women. Further, being a cluster design and taking a design effect of 2, a total of 9000 women aged 18-40 years will be studied from 10 centers (ie, 1000 per center, except for the 2 centers in Hyderabad, which have been designated with 500 each), half from a rural community and half from an urban community at each center.

Based on a pilot study previously conducted by the chief coordinator using a similar questionnaire [34], 30% (2700/9000) of the women are expected to screen positive (likely to have PCOS) on the screening questionnaire and therefore are required to be investigated in detail. Additionally, of the 70% (6300/9000) of women expected to screen negative on the screening questionnaire, 20% (1260/6300) will require examination and investigation in detail. This will make the sample size of women requiring detailed evaluation and blood testing a total of 3960 (N=2700+1260). Keeping in view the number of centers (N=10), each participant center will screen approximately 1000 women, and a total of 440 women who will be invited for detailed examinations: 20% (140/700) of screen-negative women and all 30% (300/1000) who screen positive (Figure 2).

### Evaluation Methods

Both the subgroups of women coming from the community pool and institutional pool will be evaluated as per the defined protocol for clinical, biochemical, and hormonal assessment during the second to the seventh day of the cycle. In the institutional pool, women attending endocrinology, gynecology, dermatology, or other related clinics for symptoms and signs of PCOS will be asked to report to the research staff or PCOS station for detailed evaluation as per the common protocol. Support will be obtained from the public health system for enrolling women from the community and conducting further examinations, investigations, and follow-up at their facilities in urban and rural areas. All those women who are drug-naïve will undergo clinical assessment (anthropometry, hirsutism scoring, acne, oligomenorrhea, androgenic alopecia, primary infertility), biochemical evaluation (liver function test, kidney function test, lipids, oral glucose tolerance test [OGTT]), high-sensitivity C-reactive protein (hsCRP) and cytokine tests (selected cases), 25-hydroxyvitamin D [25OHD] testing (selected cases), and hormonal estimations (thyroxine [T_4_], thyrotropin, luteinizing hormone [LH], follicle-stimulating hormone [FSH], prolactin [PRL], cortisol, anti-Müllerian hormone [AMH], dehydroepiandrosterone sulfate [DHEAS], 17-hydroxyprogesterone [17OHP], sex hormone–binding globulin [SHBG], serum total testosterone and insulin at 0, 30, and 120 minutes, estradiol [E2], progesterone [P4], procollagen type I N-terminal propeptide, CrossLaps, anti–thyroid peroxidase, ferritin, B_12_, pro–brain natriuretic peptide, and C-peptide levels). The blood samples will be transported in a cold chain to one center (SKIMS, Srinagar) for lab analysis using electrochemiluminescence immunoassay (ECLIA; Roche Diagnostics USA). Analytes such as SHBG and serum total testosterone levels will be transported to one center (SKIMS, Srinagar) for purposes of uniformity and will be analyzed in one go. In a subset of cases (n=100), total testosterone and SHBG will be assayed by ECLIA and liquid chromatography–tandem mass spectrometry in the Department of Endocrinology, AIIMS, New Delhi.

**Figure 3:**
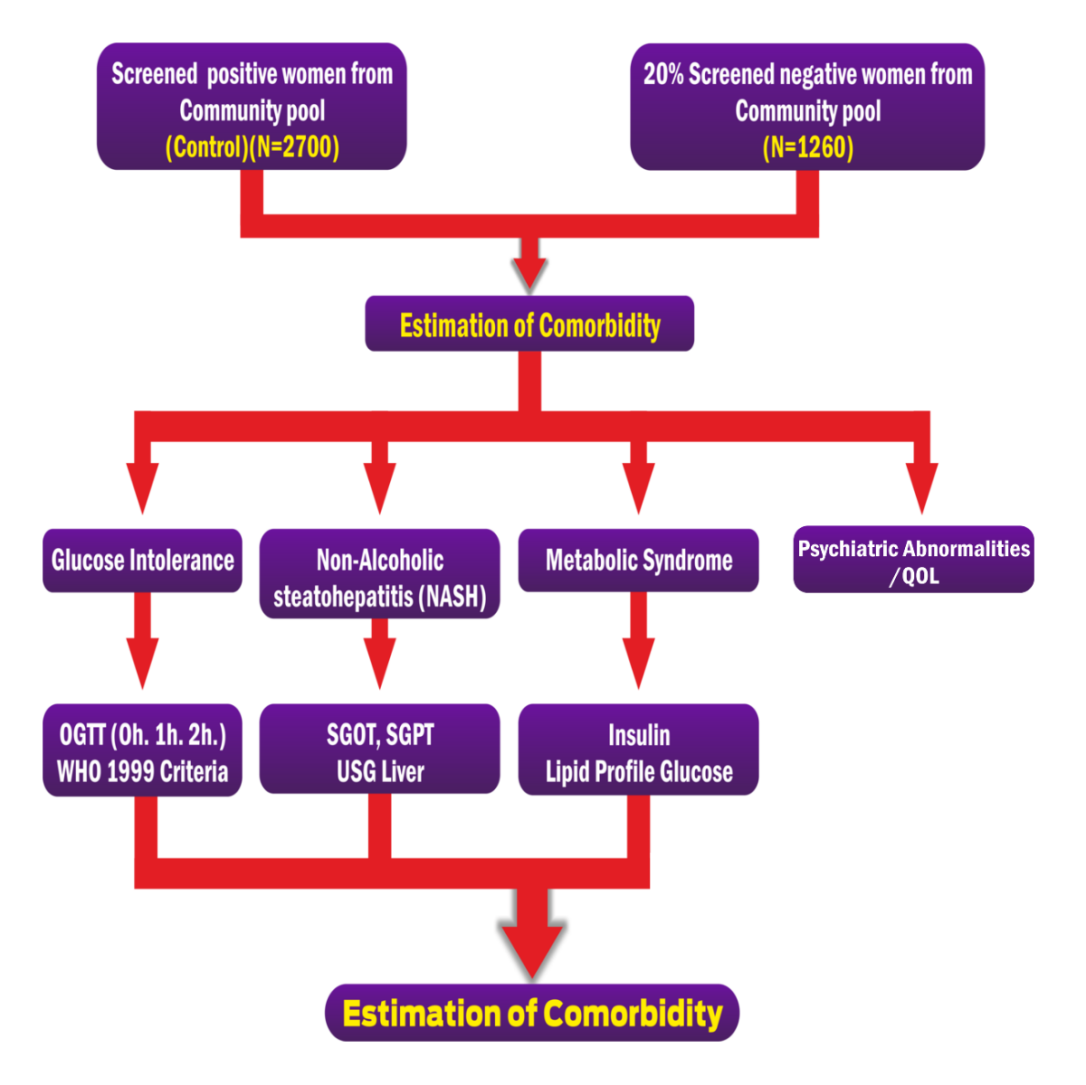
Study design and workflow chart of substudy 2. OGTT: oral glucose tolerance test; SGOT: serum glutamic oxaloacetic transaminase; SGPT: serum glutamic pyruvic transaminase; QOL: quality of life; USG: ultrasonography; WHO: World Health Organization.

The 1990 National Institutes of Health (NIH) consensus conference criteria will be taken as the qualifying criteria. This necessitates the presence of the following 2 criteria: (1) oligoovulation or anovulation (intermenstrual interval ≥35 days, presence of ≤8 cycles per year, or both) and (2) clinical signs, biochemical evidence, or both of hyperandrogenism after ruling out disorders with similar clinical presentation such as hyperprolactinemia, Cushing syndrome, congenital adrenal hyperplasia, and androgen-secreting tumors with specific laboratory analysis (cortisol, 17OHP, and DHEAS). To compare, the 2003 Rotterdam criteria and the 2009 Androgen Excess and PCOS (AE-PCOS) Society criteria will also be employed. Therefore, US examination to satisfy the diagnosis of PCOS by these criteria will be done. A common standard operating procedure for abdominal examination (liver, pancreas, ovary, and uterus) will be adhered to with a built-in system to minimize interobserver and equipment variations. The 2003 Rotterdam criteria necessitate the presence of 2 of the following 3 criteria: (1) oligoovulation, anovulation, or both; (2) clinical signs, biochemical evidence, or both of hyperandrogenism; and (3) polycystic ovary in US. The 2009 AE-PCOS Society criteria necessitate the presence of the following criteria: (1) oligoovulation and/or anovulation (intermenstrual interval ≥35 days, presence of ≤8 cycles per year, or both) and/or polycystic ovary in US and (2) clinical signs, biochemical evidence, or both of hyperandrogenism. To exclude etiologies that could mimic PCOS like Cushing syndrome, late-onset adrenal hyperplasia, or androgen-producing neoplasm, we will exclude thyroid disorders, renal hyperplasia, hyperprolactinemia, and late-onset congenital adrenal hyperplasia by medical history and hormone tests. This will further serve to unravel various comorbidities such as AGT, metabolic syndrome, IR, cardiovascular risk, psychiatric morbidities, and impairment of QOL (Figure 3). Besides, these women will be evaluated for risk factors such as birth weight, parental history of gestational diabetes or PCOS, risk of low birth weight, precocious pubarche, prepubertal obesity, antiepileptic drug intake, familial association with lifestyle diseases, etc. Abdominal MRI for comparing ovarian morphology will also be done at a selected center (SKIMS, Srinagar) using the predesigned uniform protocol.

### Study Tool: The Questionnaire

In this study, structured questionnaires will be used for capturing information. The eligibility and screening questionnaires comprise 7 and 43 questions (part 1), respectively. The screening questionnaire comprises three sections: (1) personal details, (2) menstrual history and clinical information, and (3) history of any systemic diseases. Part 1 of the questionnaires will be filled at the field site to check eligibility and identify probable PCOS on the basis of positive responses to questions. The subjects who test positive on the questionnaire (ie, probable PCOS) and 20% of those who test negative (healthy women) will be invited to the respective institutes for detailed clinical assessment. The questionnaire stands pilot-tested and modified after several rounds of investigator meetings.

### Clinical Assessment

#### History

The subjects will be invited to nearby institutes for detailed clinical, biochemical, hormonal, and sonography assessments. These women will be assessed for clinical detail about menstruation (regularity, duration, flow, amenorrhea), weight gain, unwanted male pattern hair growth, hair fall, infertility, etc. The details about any illness will be recorded. Psychiatric morbidity will be assessed using the *Diagnostic and Statistical Manual of Mental Disorders* (Fourth Edition) criteria with the Mini-International Neuropsychiatric Interview, version 4.0. The details of family pedigree will also be recorded in the predesigned questionnaire.

#### Examination

Anthropometric assessment including measurement of height, weight, waist circumference, skinfold thickness on dorsum of hand and triceps, and blood pressure and detailed systemic examination will be undertaken. Height and weight will be measured with subjects in light clothes and without shoes, using standard apparatus. Weight will be measured to the nearest 0.1 kg on a calibrated digital scale. Height will be measured by stadiometer by standard methodology to the nearest 0.1 cm. Waist and hip circumference will be measured to the nearest 0.5 cm with a measuring tape. The waist circumference will be defined as the point midway between the iliac crest and the costal margins (lower ribs), while hip circumference will be defined as the widest circumference over the buttocks and below the iliac crest [35]. Body mass index (BMI) will be derived as weight in kg divided by the square of height in meters. Overweight will be defined using the Asian BMI range of 23.0-24.9 kg/m^2^, while obesity will be defined as having a BMI equal to or greater than 25 kg/m^2^ [36]. Quantitation of hirsutism by a single observer by counting 9 specific body areas for the Ferriman-Gallwey score and assessments of acne vulgaris, androgenic alopecia, and acanthosis nigricans will be performed in all subjects [37].

#### Radiological Examination

Ultrasonography of the abdomen will be performed to evaluate the polycystic ovary morphology and grading of fatty liver (NAFLD) and nonalcoholic pancreatic steatosis. The US will be performed at all centers, and MRI of the pelvis (n=100) will be performed primarily at SKIMS, Srinagar, to compare US and standardization of US technique by 2 independent radiologists. MRI will be done as per a randomized selection proportional to the subjects at the site.

#### Laboratory Evaluation

The OGTT will be performed after an overnight fast of 10-12 hours. An oral glucose load with 75 g of anhydrous glucose dissolved in 250-350 mL of water will be administered over 3-5 minutes immediately after the basal sample. The blood samples for LH, FSH, testosterone, AMH, SHBG, and DHEAS will be collected on days 3-7 (early follicular phase) of spontaneous or medroxyprogesterone-induced (in amenorrhea patients) menstrual cycle to confirm PCOS. Hematological parameters (hemoglobin, red blood cells, white blood cells, etc), biochemical tests like lipid profile, liver function, kidney function, blood glucose (OGTT), and hormonal assays like T_4_, thyrotropin, LH, FSH, PRL, E2, P4, cortisol, testosterone, AMH, DHEAS, 17OHP, and insulin at 0 hours, 30 minutes, and 2 hours (optional) will be carried out at the respective centers, and special hormones like testosterone and SHBG will be done in the central facility to be created at the coordinating center (SKIMS, Srinagar). On the day of testing, women will be called after 10-12 hours of fast, and blood samples (15 ml) will be drawn. Blood will be also collected for glucose and insulin estimation after 30 minutes and 120 minutes of 75 g oral glucose challenge. Insulin samples will be immediately put in a refrigerator, separated in cold centrifuge, and stored at −20 °C until the assay. Plasma will be separated in a refrigerated centrifuge at 1560*g* for 10 minutes, and sera will be stored at −80 °C until tested.

Plasma glucose will be measured using the glucose oxidase–peroxidase method, and hormonal tests (LH, FSH, T_4_, thyrotropin, PRL, E2, cortisol, testosterone, AMH, hsCRP, cytokines, and 25OHD) will use ECLIA on Cobas e411 (Roche Diagnostics USA).

### Methodology: Protocol for Substudy 3

**Figure 4:**
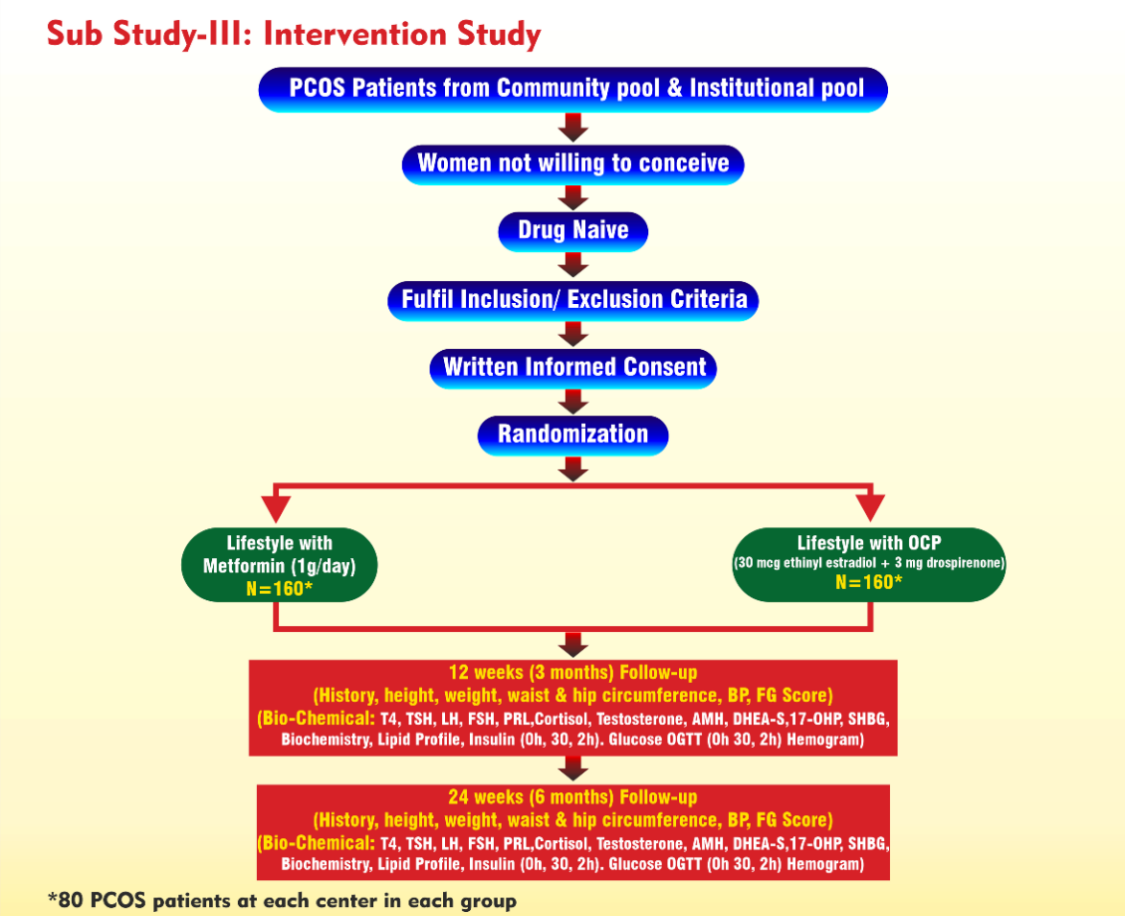
Study design and workflow chart of substudy 3. 17-OHP: 17-hydroxyprogesterone; AMH: anti-Müllerian hormone; BP: blood pressure; DHEA-S: dehydroepiandrosterone sulfate; FG: Ferriman-Gallwey; FSH: follicle-stimulating hormone; LH: luteinizing hormone; OCP: oral contraceptive pill; OGTT: oral glucose tolerance test; PCOS: polycystic ovary syndrome; PRL: prolactin; SHBG: sex hormone–binding globulin; T4: thyroxine; TSH: thyrotropin.

The study will be conducted only at 2 centers: AIIMS, New Delhi, and SKIMS, Srinagar. Women (aged 18-40 years) residing in the communities (rural and urban) identified in the prevalence study and those attending outpatient clinics in the departments of either endocrinology, gynecology, or dermatology of the selected institutes who are willing to participate will be recruited in the interventional part of the study. All these women will undergo clinical, biochemical, and hormonal assessment at baseline and at 3 and 6 months (Figure 4).

#### Sample Size Calculation for Substudy 3

On the basis of one study, metformin could improve the Homeostatic Model Assessment of IR from 2.5 (SD 0.39) to 2.79 (SD 0.39) [38]. Assuming that the baseline was similar, at an α error of 0.05 with power of 90%, and with a projected attrition rate of 20%, we need to recruit 153 women per group. To round off, we will recruit and randomize 160 women with PCOS per group (ie, a total of 320 women). A total of 160 subjects will be recruited and randomized in each of the 2 groups, with 80 subjects in each arm.

#### Randomization (Drug Intervention)

Eligible consenting subjects will be randomized in a 1:1 ratio into 2 arms through a blinding procedure.

Arm A compares lifestyle (32 kcal/kg diet per day with 55% carbohydrates, 20% protein, 20% fat, and 20 g of fiber with 30 minutes of brisk walking daily) and metformin (1 g /day) versus lifestyle alone (n=160; 80 per center).

Arm B compares lifestyle (32 kcal/kg diet/day with 55% carbohydrates, 20% protein, 20% fat, and 20 g of fiber with 30 minutes of brisk walking daily) and OCP (30 µg ethinyl estradiol plus 2 mg drospirenone) versus lifestyle alone (n=160; 80 per center).

#### Subject Selection

The subjects will be recruited from the community pool of substudy 1 and the institutional pool. The eligibility based on inclusion and the investigators will assess exclusion criteria. The institutional pool, which comprises the women attending endocrinology, gynecology, or other clinics for various complaints suggestive of PCOS, will be taken for purposes of recruitment in the intervention part of phase 1 or for further assessment in forming a long-term cohort. Potentially eligible patients will be recruited from the outpatient department services of the participating centers. If the subjects qualify on the inclusion and exclusion criteria, they will be informed about the details of the study and hence will be approached for written informed consent. All potentially eligible patients will undergo clinical, biochemical, and imaging (US) assessment to confirm PCOS. A logbook will be maintained for all patients undergo screening, randomized, eligible but not randomized along with reasons for non-randomization. For the intervention study (substudy 3), the subjects will be recruited only from AIIMS, New Delhi, and SKIMS, Srinagar. The sleep study and carotid Doppler at SKIMS and AIIMS, New Delhi, will be conducted for 100 subjects at each site.

The drug-naïve women who qualify on the 1990 NIH criteria (from both the institutional pool and the community pool) will be invited for the study. This will be an open-label randomized intervention trial and will follow a common protocol at each participating center. Women who do not desire fertility and who furnish written informed consent (second time) will undergo a detailed clinical assessment including details of menstrual, drug, and other relevant history followed by clinical examination involving anthropometry (height, weight, waist circumference); Ferriman-Gallwey scoring for hirsutism; grading of acanthosis nigricans, androgenic alopecia, and acne vulgaris (grades 1-3); blood pressure measurement; and a brief relevant systemic examination. Biochemical and hormone estimation will be done at baseline from the second day to the seventh day of the menstruation cycle and at 3 and 6 months. The subjects will be followed up at 6 months, and all these investigations will be repeated at the follow-up. A total of 320 women for the intervention phase (ie, 80 subjects per arm and per center) will be recruited. The study will be done only in 2 participating centers (SKIMS, Srinagar, and AIIMS, New Delhi). The intervention phase will be initially done for a period of 24 weeks.

#### Baseline Assessment

Standardized study tools will be used to record patient name, contact details, history, family history, personal information (age of menarche, menstrual regularity information, acne severity), food habits, lifestyle habits, previous disease information, drug intake history information, and clinical details including anthropometry (height, skinfold, waist circumference) by using anthropometric tools (SECA 213 height scale, SECA 813 weight scale, SECA 203 nonstretchable tape, and calipers measuring skinfold thickness), blood pressure (using Omron blood pressure instruments), Ferriman-Gallwey score, androgenic alopecia, acanthosis nigricans, acne vulgaris, and systemic examination. Measurement of biochemical parameters (blood glucose, OGTT, lipid profile, liver function, kidney function), protein:creatinine ratio, and 25OHD (in selected cases); hormonal assays (like T_4_, thyrotropin, LH, FSH, PRL, E2, P4—21st day optional, cortisol, testosterone, AMH, DHEAS, 17OHP, SHBG, and insulin); and radiological assessment will be done at baseline and after 6 months of treatment.

#### Follow-Up and Safety Evaluation

Patients will be evaluated for any adverse events and efficacy at 90 days (−7 days to +7 days) and 180 days (−7 days to +7 days) through follow-up. All clinical and biochemical investigation will be performed in a similar fashion as done at baseline. If the patient is not available for assessment at 180 days (−7 days to +7 days) follow-up, this will be considered a protocol deviation. The safety of different interventions will be assessed by recording the development of immediate or delayed complications recorded in a form with a prespecified list of safety parameters. Immediate reactions will include allergic reactions, diarrhea, vomiting, deep vein thrombosis, etc in a prespecified checklist.

#### Field Activities

Field activities will be conducted for the purposes of screening. It will include rapport building among locals and the selection of the study subjects (1 woman per household). It will be the duty of the research staff to approach the selected individuals and to make them understand their role in the study. Consenting individuals will be screened for PCOS phenotype using the predesigned structured questionnaire. The screening questionnaire will be used to obtain information on personal identification details, demographics, socioeconomic status, menstrual history and clinical information, history of any systemic disease, and QOL. The women who have hyperandrogenism or oligomenorrhea with or without obesity and acanthosis nigricans (IR) will be taken as probable cases of PCOS. Screen-positive and selected screen-negative (regular menstrual cycles, no features of hyperandrogenism or insulin resistance) individuals will be informed to visit the hospital for further investigations.

### Statistical Analysis

Data will be collected on predesigned proformas. The data obtained will then be entered using pilot-tested CSPro software (version 6.2; United States Census Bureau). After assessing for approximate normal distribution, all continuous variables will be summarized as mean (SD) or median (IQR). Categorical variables will be expressed as n (%). Student *t* test and analysis of variance will be used to compare the groups. Statistical analysis will be performed using reliable SPSS statistical software (version 26; IBM Corp).

## Results

The study was ethically approved and funded in November 2017 by ICMR, New Delhi, India. The subject recruitment from the community pool and implementation of the intervention for evaluating the treatment responses in a subset of PCOS subjects started in 2018 and is expected to be completed in October 2021. The study results will be compiled and statistically analyzed by April 2022 and will be published in a peer-reviewed scientific journal.

## Discussion

Polycystic ovary syndrome is a multifactorial disorder with unknown etiology and numerous clinical manifestations in reproductive-aged women around the world [4-7]. The diagnostic criteria used to define PCOS include menstrual irregularity, hyperandrogenism, and polycystic ovary morphology. To date, PCOS has remained a major clinical challenge due to its poor prognosis, limited treatment options, and late diagnosis of the disease.

The prevalence of PCOS is increasing the world over and is showing a galloping increase in India. To date, there have been no systematic studies available either globally or in India. In India, there are undersized, convenience-based studies reported, which might not reflect the true status of PCOS prevalence in the community [32,33]. It is difficult to draw a clear conclusion from this limited number of studies conducted across the country. These studies hint at the potential for large-scale epidemiological studies on PCOS across India. This ongoing study will be the first of its kind in the country to generate the actual prevalence and picture of the disease in the community.

It is the first nationwide initiative by ICMR, New Delhi, to comprehensively study the prevalence, comorbidities (obesity, AGT, IR, NAFLD, sleep apnea, stroke, CVD, neuropsychiatric comorbidities), and phenotypes of PCOS in India using robust diagnostic methodology. On its completion, the study will generate an unprecedented amount of data about PCOS and the efficacy of interventions, which will facilitate the health care professionals, policy makers, and government of India to formulate new strategies that may shift the existing treatment paradigm in the near future.

## Appendix

Multimedia Appendix 1 ICMR-PCOS Study-Expert committee recommendations.

## Abbreviations

17OHP: 17-hydroxyprogesterone
25OHD: 25-hydroxyvitamin D
AE-PCOS: Androgen Excess and Polycystic Ovary Syndrome
AGT: abnormal glucose tolerance
AIIMS: All India Institute of Medical Sciences
AMH: anti-Müllerian hormone
BMI: body mass index
CVD: cardiovascular diseases
DHEAS: dehydroepiandrosterone sulfate
E2: estradiol
ECLIA: electrochemiluminescence assay
FSH: follicle-stimulating hormone
hsCRP: high-sensitivity C-reactive protein
ICMR: Indian Council of Medical Research
IR: insulin resistance
LH: luteinizing hormone
MRI: magnetic resonance imaging
NAFLD: nonalcoholic fatty liver disease
NIH: National Institutes of Health
OCP: oral contraceptive pill
OGTT: oral glucose tolerance test
P4: progesterone
PCOS: polycystic ovary syndrome
PRL: prolactin
QOL: quality of life
SHBG: sex hormone–binding globulin
T_4_: thyroxine
US: ultrasonography; ultrasound
