# Supplementary material for: Evaluation of the Prevalence, Regional Phenotypic Variation, Comorbidities, Risk Factors, and Variations in Response to Different Therapeutic Modalities Among Indian Women: Proposal for the Indian Council of Medical Research–Polycystic Ovary Syndrome (ICMR–PCOS) Study
Source: JMIR Res Protoc. 2021 Aug 27;10(8):e23437. doi: 10.2196/23437 (PMC8433859; doi:10.2196/23437)
Supplement: Multimedia Appendix 1 [file resprot_v10i8e23437_app1.pdf]

Minutes for integrated project on "Evaluation of prevalence, regional phenotypic variation, comorbidities and risk factors among Indian women with polycystic ovary syndrome (PCOS) and the variation in response to different therapeutic modalities: A Multicenter study across India" (An ICMR Task Force Project) held on May 17, 2016 at ICMR Hqrs., New Delhi.

The following attended the meeting:

Experts

1. Dr. Kameshwar Prasad, Deptt of Neurology, AIIMS, New Delhi
2. Dr. Y.K. Gupta, Deptt of Pharmacology, AIIMS, New Delhi
3. Dr. Rajesh Sagar, Deptt of Psychiatry, AIIMS, New Delhi

ICMR Secretariat

8. Dr. R.S Sharma, Head (RBMH)
9. Dr. N. Chandhiok, Scientist 'G'

Based on the suggestions given by the experts and the ensuing discussion, the following recommendations were made

- **Criteria for inclusion of participating Centre / Institute** should be objective and based on the interest envisaged by the Site principal Investigator to participate in the study, the capacity of the site to recruit the sample size in the stipulated period, it should have mandatory equipments for diagnosis and place for storage of blood samples and it should have a rural base from where subjects could be enrolled. ICMR coordinating site would make site visits wherever necessary to confirm the suitability of the site prior to the initiation of the study.

- **Diagnostic criteria for PCOS:** Dr. K Prasad suggested that as there is no gold standard for PCOS, the diagnostic criteria for PCOS should be in accordance with that suggested by NICHD, 1990. The Rotterdam, 2003 and AEPCOS Society, 2009 criteria may cause variation in the data collection due to variation in USG data capturing. Therefore, TVS as a mandatory investigation may be excluded.
- Diagnosis of PCO is more accurate using MRI. If possible, at some centre(s), both MRI and USG should be done instead of USG alone using a specified protocol. The film should be analysed at one centre (by independent team of two investigators) and the **correlation of between USG and MRI in diagnosis of PCO studied.**
- The **sample size of 9000 (1000/Centre) is adequate** to study the objectives of the study.
- **Testosterone estimation** should be done using **LC/MS**. Since, this facility is not available at all sites, it should be done at one site centrally where the blood sample should be sent. It was suggested that collaboration can be explored with National Dope Testing Laboratory in Delhi where this facility is available under the supervision of Dr. Alka Butra.
- Dr. Y.K. Gupta suggested that an **Indian scoring algorithm** for diagnosis of PCOS can be developed by giving different weight age to clinical, biochemical and radiological criteria. On basis of S/S, a case can be defined as provisional PCOD. A final assessment would be made by coordinating lab to confirm the case as a definitive.
- The **sub study III (Intervention Study)** would be carried out only at the selected participating centres. The sub study-III will be done with two arm study, **Group (I)** Lifestyle (32 kacal/kg diet/day with 55 % carbohydrates, 20% protein, 20% fat and 20 grams fibre with 30 minutes brisk walk daily) alone with Metformin (1g /day) and **Group (II)** Lifestyle with OCP (30 mcg ethinyl estradiol plus 3mg drospirenone). The protocol for sub studyIII (intervention study) should be design in accordance with the clinical trial protocol.

To coordinate all the activities of the Task Force study and to fix-up the responsibilities of various stack holders, discussions were held on 19th July, 2016 among Dr. R. S. Sharma, Head-RBMH; Dr. Nomita Chandiok, Sci-G, Co-ordinatior ICMR; Dr. Mohd Ashraf Ganie, Chief Co-ordinatior cum Principal Investigator;

Minutes of the Investigators Meeting for the Task Force Project entitled Evaluation of prevalence, regional phenotypic variation, comorbidities, risk factors and the variations in response to different therapeutic modalities among Indian women with polycystic ovary syndrome (PCOS): A Multicenter study across India" held on 11th May 2017 at SKICC, Srinagar during 2nd J&K Medical Science Congress & 1st Annual Conference of M P PCOS Society, 10-13 May 2017.

Dr. R S Sharma, Head RBMH ICMR, New Delhi Chaired the meeting. He welcomed all the members and also introduced the purpose and plan of the TASK Force study. Dr. Mohd Ashraf Ganie, Project Coordinator, put the current status of the project as follows:

- The project has been modified as per the Dr. Dash suggestions.

- As per ICMR, New Delhi requirement, each centre is requested to complete the Codal formalities which include Declaration & Attestation, Undertakings, Ethics Committee approval, Mandate Form – extra mural grants (Name of the Statutory Auditors of the host Instt. along with complete address) etc.

- The current status of Codal formalities was informed that all centres have provided codal formalities except GMC, Thiruvananthapurm. Dr. Mohd Ashraf Ganie informed that all codal formalities from GMC, Thiruvananthapurm will be completed within one two week, as per discussion with Dr. P K Jabbar (PI).

- Dr. Mohd Ashraf Ganie has informed that the ultrasound will be performed at each centre in all subjects with a uniform protocol. For purposess of uniformity sonologits have to grill on equipments, probe suitability and method od examination, MRI will be performed only at SKIMS, Srinagar and AIIMS New Delhi (100 subjects each).

Minutes of the Monitoring cum Expert Committee meeting of the ICMR Task Force study on PCOS held on 12.5.2018 at ICMR Hqrs, New Delhi

The following members attended the meeting

1. Dr. RandeepGuleria, Director AIIMS, New Delhi - **Chair**
2. Dr. Kameshwar Prasad, Prof. & Head, Dept of Neurology, AIIMS, New Delhi- **Co-Chair**
   Members
3. Dr. S.V. Madhu, Director-Professor& Head, Dept. Of Endocrinology, University College Of Medical Sciences and GTB Hospital, Delhi-**Co-Chair***
4. Dr. Vishnu VardhanaRao, Director National Institute of Medical Statistics, New Delhi
5. Dr. Surinder Singh, Director, National Institute of Biologicals, New Delhi
6. Dr. AlkaKriplani, Professor and Head, Dept of Obstetrician & Gynecologist AIIMS, New Delhi
7. Dr. A. B. Dey, Professor & Head Department of Geriatric Medicine AIIMS, New Delhi
8. Dr. Sarita Bajaj, Director-Professor & Head, Dept of Medicine MLN Medical College, Allahabad #
9. Dr. Y. K. Gupta, Prof. & Head Department of Pharmacology AIIMS, New Delhi *
10. Dr. S. K. Singh, Professor, Department of Endocrinology Banaras Hindu University,UP*
11. Dr. PuneetMisra, Professor, Department of Community medicine, AIIMS, New Delhi
12. Dr. Mohd Sultan Khuroo, Chairman Digestive diseases Center & Former Director SKIMS Srinagar *

   Coordinating Team

13. Dr. R S Sharma, Scientist G and Head RBMH, ICMR HQrs
14. Dr. Mohd Ashraf Ganie Chief Coordinator and Principal Investigator.
15. Dr. Shalini Singh Scientist F, ICMR HQrs, New Delhi,

Prof R Guleriastarted his opening remarks by emphasizing the magnitude of PCOS as public health problem that has gradual onset and has varied manifestations. He highlighted its presentations across specialties and therefore hinted at the need of members from different specialties of medicine to steer this very important project. He informed that the project has been developed over a long time and hoped that with disbursement of funds the process of subject enrollment would begin soon. He applauded and then requested Dr. Ashraf Ganie to present the study protocolin detail for deliberations.

Dr. Ashraf Ganie provided a detailed presentation on the study background, objectives, design, methodology including sampling technique and sample size. He explained that the sample size is calculated to provide only national prevalence of PCOS and checking comorbidities and interventional study at two sites only. He also elucidated in detailthe implementation strategy both at the community level and institutional level. He explained the procedure of capturing data at the community level using a questionnaire followed by institutional work up to record medical history, clinical examination and lab procedures. He cautioned about the fallacies of current ways of diagnosing PCOS and capturing data. He gave a detailed plan to address these issues particularly adopting uniform procedures for anthropometry and laboratory tests including haemogram, biochemistry and hormonal assays and ultrasonography. Regarding screening questionnaire Dr. Ashraf informed that it has been made as inclusive as possible so that the chances of missing out a case are minimal. Responding to the question of sensitivity of the questionnaire, Dr. Ashraf informed that piloting has been already done and is being validated. Dr. Ashraf also informed that PCOS being a diagnosis of exclusion the proforma cannot be highly specific but has to be sensitive. The biochemistry will be done by respective centers using automated systems, however, hormones will be transported to central laboratory set at coordinating center (SKIMS) using high end system of Electrochemiluminescence (ECLIA) technique with good sensitivity and specificity. Serum total testosterone and SHBG will be assayed using LCMS/MS at AIIMS New Delhi in a select set of samples. He further informed that USG will be done by same sonologists (CO-PIs already trained in the workshop) on the same machine at the respective centers for which inter-observer variability is beingcurrently done by blind assessment of circulated images. In case the variability is more than 7-10% retraining will be done. Dr. Ashraf also mentioned the challenges and sought suggestions and guidanceof the committee to address these issues at the earliest. In the end Dr. Ashraf asked for support of phase II of the study where identifying risk factors, doing novel interventions and maintaining long-term cohorts is proposed.

Minutes of Meeting of the Monitoring cum Expert Committee for the Task Force Project entitled "Evaluation of prevalence, regional phenotypic variation, comorbidities, risk factors and the variations in response to different therapeutic modalities among Indian women with polycystic ovary syndrome (PCOS): A Multicenter study across India" held on 16th Jan 2019 at ICMR Headquarter, New Delhi

Following members attended the meeting:

1. Dr. Kameshwar Prasad, Prof & Head Deptt. of Neurology AIIMS, New Delhi (Co-Chair)
2. Dr. S V Madhu, Prof & Head Deptt. of Endocrinolgy GTB Hospital, N Delhi
3. Dr. AB Dey, Professor and Head Deaprtmnt of Geriatrics AIIMS, New Delhi.
4. Dr. Alka Kriplani, Ex-Professor &Head, Obstetrics & Gynecology AIIMS, N Delhi.
5. Dr. Surinder Singh, Director National Institute of Biolgicals. New Delhi.
6. Dr. Neerja Batla, Professor Obstetrics and Gynecology AIIMS, New Delhi.
7. Dr. Sarita Bajaj, Professor and Head, Dept of Medicine, GMC, Allahabad, (through Skype).
8. Mr. B S Dhillon, Sci G, NIMS New Delhi. (in lieu of Dr MMV Rao, Director NIMS)

Coordination Centre

1. Dr. R S Sharma, Scientist G and Head RBMH ICMR Head quarters, New Delhi.
2. Dr. Mohd Ashraf Ganie, Professor of Endocrinology SKIMS, Srinagar (Chief coordinator)
3. Dr. Shalini Singh, Scientist 'F' RBMH ICMR Head quarters, New Delhi

Dr. R S Sharma, ICMR, New Delhi wellcomed all the members and gave a brief outline about the importance of the study and the purpose of the meeting. He invited Prof. Kameshwar Prasad to preside over the meeting as per the communication of Prof Randeep Guleria (Chair).

Dr. Kameshwar Prasad introduced the members and lauded the efforts of ICMR and Chief Coordinator for initiating such an important study. He invited Prof. Mohd Ashraf Ganie, project Coordinator to give a presenation about the milestones covered in the first year of the study.

Dr. Ashraf Ganie presented the annual report in detail. This included holding of Investigators' meetings to finalize study protocol and study tools; recruitment and training of the staff, procuremnt of equipments and consumables; and initiation of field surveys and investigations at all sites. He apprised the Committee about the challenges faced during implementation of the project and the solutions worked out. He mentioned about the setting up of a single centre for hormone estimation with dedicated analyser and sample transport system; procedure of training and certification of Junior Medical Officers (JMOs) and working out the agreement of observations between the sonologists of all the sites. Members appreciated the adoption of this methodology as it will add strength to the study. He however advised the PI to provide raw data and interclass correlation coefficient of all observations obtained in JMOs testing and certification. He said this will enhance the robustness of data. Dr. S V Madhu along with other members suggested that during the field surveys data about reasons of ineligibilty of subjects should be obatined. Dr. Alka Kriplani showed concern about the "Known PCOS" category in study tool. It was discussed that the label of 'known

19/11/19

Prof. K. Prasad
Head, Deptt. of Neurology &
Chief Neurosciences Centre
[illegible]Delhi-110029

Minutes of the Monitoring-cum-Expert Committee Meeting for the ICMR Task Force study held on 22nd Oct. 2019 at 11:00 AM at Conference Hall, Room no. 301, ICMR Hqrs, New Delhi

After deliberations on the agenda among all the members a consensus was achieved with due approval by the chair and following recommendations were made:

Recommendations

1. The Committee recommended the continuation of the study to the 3rd year, with the additional budget, discussed at point no. 3 and 5.
2. Committee asked the chief coordinator and all PIs to expedite the study so that the study can be completed within the given timeline.
3. The committee asked Chief Coordinator to build a system for periodic surveillance of data. It was recommended that SKIMS should appoint a Scientist B (non-medical) for monitoring/auditing of data and sites. The monitor/auditor should be given the task of
    a. Weekly checking of data (Every Friday)
    b. Inspection visits to study sites along with the other members of auditing/monitoring committee.

[illegible]

Department of Endocrinology
Sher-i-Kashmir Institute of Medical Sciences (SKIMS)
Srinagar, J&K, India

Dr. Mohd Ashraf Ganie, MBBS, MD, DM
Professor
Editor J of Diabetes and Obesity, International Journal
Associate Editor IJEM
President M P PCOS Society

To **Date: 10/02/2017**

Dr. R. S. Sharma
Head, RBMH & CH
ICMR, New Delhi
Attention: Dr. Nomita Chandhiok, Consultant, RBMH Division, ICMR, New Delhi

Sub: Evaluation of project entitled "**Evaluation of prevalence, regional phenotypic variation, comorbidities, risk factors and the variations in response to different therapeutic modalities among Indian women with polycystic ovary syndrome (PCOS): A Multicenter study across India"**

Dear Dr. Sharma,
We are thankful to you and Prof. R.J. Dash for a constructive review of the above mentioned proposal. Kindly find the modified proposal in response of comments given by the Chairman. Our point by point response to the comments of respected Chairman are as follows:

Comment 1: It appears to be an ambitious project, with loaded objectives.
Reply: We highly appreciate the positive appraisal made by the respected reviewer and also for approving the modifications done in response to the comments of previous PRG.

Comment 2: It should initially focus on (1) estimating prevalence and (2) study phenotypic variations of PCOS in different population groups in the country, if any.
The prevalence study should include population groups from
Kashmiris, Dogras, Sikhs and Jats, Rajputs, western UP in North; Interiors of Maharastra, Gujratis in Gujrat, and Goans in West; Central MP, Tribals from Jharkhand, Odisha and Chhattishgarh in Central India; Telugus, Tamils, Kannadigas (Karnatakis) and Keralites in South; Bengalis in East and native people of North-east including tribals in North-east in phases.
Accordingly, Centers should conveniently be located having services/experts in Endocrinology, Gynecology and Radiology /Sonographist at the least, *with proven interest in PCOS.*
Reply: The comments of the Chairman are very valuable with regard to studying prevalence/phenotypic variation in the study. To address the issue of phenotypic variation, we are keen to include all these regional/ethnic groups. This will include roping in 9 more centers with appropriate facilities and the experts in the related specialties (gynecology, radiology and endocrinology) with a proven track record of clinical and research experience in the area of PCOS. We request the funding agency to enhance the grant for this part of the study. However, for time being we propose to initiate the 10 identified centers and subsequently rope in the nine more centers. It may be worthwhile to mention here that initially the selection of centers was based on some criteria such as:

- ❖ The experts working or having interest in the area of PCOS.
- ❖ Centers having requisite infrastructure
- ❖ Centers representing a zone or ethnic population

Correspondence: Department of Endocrinology, Sher - i - Kashmir Institute of Medical Sciences
Soura, Srinagar, Jammu & Kashmir, India - 190011
Mobile No: +91-194-2402418; 91- 9419041546; Fax: 91 - 194 - 2403470
Email:- asharafendo@rediffmail.com / ashraf.endo@gmail.com

Comment 3: The field staff engaged for prevalence study at different centers should undergo an orientation program to understand various terminologies as apply to menstrual disorders, grading of hirsutism and features of hyper-androgenism etc. The project I/C on prevalence study component should also periodically review findings of field staff at various locations.
Reply: The plan of orientation program for the investigators and field staffs are involved in the study has already been proposed. The periodic review of the data capturing the methods employed etc. have been incorporated in the project proposal as suggested. (Page-68)

Comment 4: For study of (2) phenotypic variations, Criteria for diagnosis of PCOS should include all components as envisaged by NIH (1990), Rotterdam (2003) and AE-PCO Society (2009). This will provide scope to determine % variation in diagnosis with one or the other criteria in Indian context. Phenotypic features to look for should be listed in the study pro-forma.
Reply: This is a very valid advice and we will try to employ all the three sets of criteria for diagnoses. However, it will need procuring Ultrasound machines at the centers where it is not available and also conducting ultrasound training workshops for sonologists to minimize inter and intra observer variations.

Comment 5: The preliminaries for the study: (1) Correlation between pelvic/IV US with pelvic MRI for ovary in small number of cases (say 25) (2) Testosterone estimation by clinical assays and LCMS in few samples (say 25-30). (3) CV of hormone estimates from sample storage and transport and (4) External QC for different hormones and biochemical parameters between participating centers should be addressed early during project period.
Reply:
(1) The correlation between pelvic/intra vaginal ultrasound with pelvic MRI is already planned at two centers i.e. SKIMS, Srinagar and AIIMS, New Delhi.
(2) Testosterone estimation by LC/MS is already planned for selected 100 samples.
(3) CV of hormone estimates from sample storage and transport is incorporated in the proposal.
(4) External QC for different hormones and biochemical parameters between participating centers is also addressed (page-61).

Comment 6: Assessment of co-morbidities and specific treatment benefits should be left to discretion of senior investigators of advanced centers, and subjected to review as the project proceeds.
Reply: The assessment of comorbidities, treatment benefits including OCP's vs. Metformin (randomized control trial) will be done at two centers (SKIMS, Srinagar & AIIMS, New Delhi) as suggested (page-76).

Comment 7: The suggestions made above may be addressed to. Mechanism for strict academic vigil should also be built-in for periodic review.
Reply: A built-in mechanism for periodic review for academic vigil is incorporated as suggested (page-70).

Thanking you

Dr. Mohd Ashraf Ganie
Professor, Department of Endocrinology
Project Coordinator
SKIMS, Srinagar

Prof. Mohd Ashraf Ganie
Chief Coordinator
ICMR PCOS Task Force

Training cum Certification of JMO's (Junior Medical Officers) from 10 study sites for ICMR-PCOS Task Force study: Evaluation of prevalence, regional phenotypic variation, co morbidities, risk factors and variations in response to different therapeutic modalities among Indian women with polycystic ovarian syndrome (PCOS): Multicentre study across India jointly organized by Sher-i-Kashmir Institute of Medical Sciences [SKIMS] and ICMR at SKIMS Board Room on 19th and 20th November.

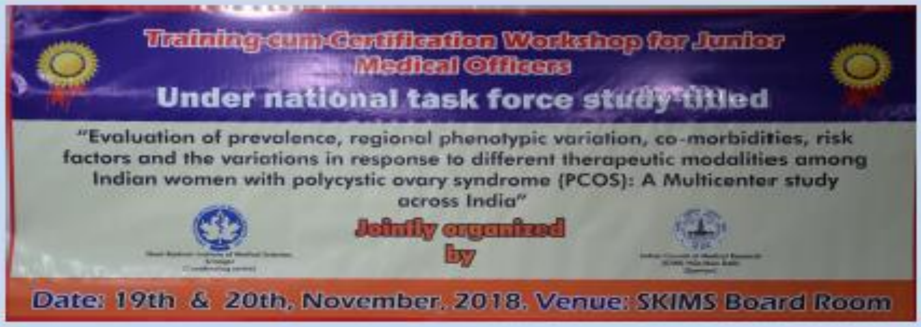

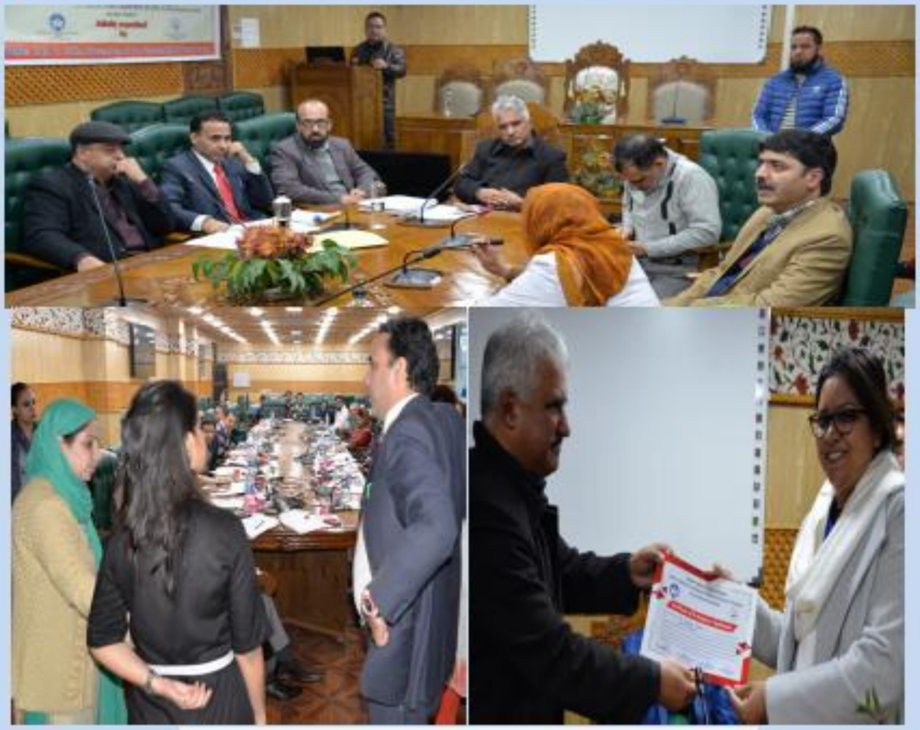

ICMR-PCOS Task Force Study
Training cum Certification workshop
